# Leaf functional traits and their effects on photosynthetic carbon fixation of eight deciduous shrubs in urban parks

**DOI:** 10.3389/fpls.2026.1814790

**Published:** 2026-06-26

**Authors:** Han Li, Sidong Shen, Jing Qian, Xiaolu Li, Yitong Li, Li Dong, Shuxin Fan

**Affiliations:** 1 College of Landscape Architecture, Beijing Forestry University, Beijing, China; 2 Laboratory of Beijing Urban and Rural Ecological Environment, Beijing, China; 3 National Engineering Research Center for Floriculture, Beijing, China; 4 Guangxi Forest Inventory and Planning Institute, Nanning, China; 5 Taicang City Chengrong Investment Limited Company, Suzhou, China

**Keywords:** landscape plants, leaf economics spectrum, leaf functional traits, photosynthetic carbon fixation, trade-off relationships

## Abstract

**Introduction:**

Urban vegetation plays a crucial role in enhancing carbon sinks. However, how leaf functional traits affect photosynthetic carbon fixation in landscape deciduous shrubs remains unclear. Identifying key leaf functional traits indicative of high carbon fixation is essential for selecting and breeding landscape shrubs with high carbon fixation capacity.

**Methods:**

This study investigated the leaf structural, chemical, and physiological traits, leaf lifespan, and photosynthetic carbon fixation of eight common deciduous shrubs in urban parks, including *Hibiscus syriacus*, *Rosa hybrida*, *Sorbaria sorbifolia*, *Deutzia parviflora*, *Cornus alba*, *Viburnum opulus subsp*. *calvescens*, *Lonicera maackii*, and *Weigela florida*. Pearson correlation analysis, principal component analysis (PCA), and partial least squares (PLS) regression were used to explore leaf functional traits and their effects on photosynthetic carbon fixation.

**Results:**

The results showed that *H. syriacus*, *R. hybrida, C. alba*, and *W. florida* exhibited higher specific leaf area, relative chlorophyll content, and stomatal conductance, demonstrating stronger carbon fixation capacity. In contrast, *S. sorbifolia*, *D. parviflora*, *V. opulus*, and *L. maackii* showed higher leaf mass per area and leaf dry matter content, as well as longer leaf lifespan, exhibiting weaker carbon fixation. High carbon fixation capacity of plants is an integrated outcome of covarying traits, including high values of specific leaf area, leaf nitrogen and phosphorus content per unit area, relative chlorophyll content, stomatal conductance, transpiration rate, and photosynthetic nitrogen use efficiency. This trait combination trades off with higher leaf mass per area, higher leaf dry matter content, and longer leaf lifespan. Specific leaf area, relative chlorophyll content, and leaf lifespan were identified as the key leaf functional traits affecting photosynthetic carbon fixation in plants.

**Discussion:**

Our findings revealed that leaf functional traits collectively regulated photosynthetic efficiency and annual carbon assimilation through synergies and trade-offs, thereby influencing photosynthetic carbon fixation. This study provide a bioecological basis for selecting and breeding landscape shrubs with high carbon fixation.

## 1 Introduction

The rapid pace of urbanization and industrialization has led to continuous increases in CO_2_ and other greenhouse gases, intensifying climate change and associated extreme weather events (Fischer et al., 2021; Quilcaille et al., 2025; Wake, 2023; Walsh et al., 2020). This poses serious challenges to urban ecological security, human living environments, and sustainable socioeconomic development. As a critical natural element within urban ecosystems, urban vegetation serves not only as an important medium for mitigating the urban heat island effect and improving air quality, but also as the primary natural carbon sink in urban areas (Qian et al., 2024). Its ecological value is becoming increasingly prominent. Traditional urban greening designs have often prioritized aesthetic and ornamental values. However, under the dual pressures of climate change and urbanization, a central research challenge has emerged: how to scientifically select and configure tree species that combine landscape functions with high carbon fixation capacity and strong environmental adaptability, thereby enhancing the overall carbon sink function and ecosystem service efficiency of urban vegetation.

Plants in urban green spaces convert atmospheric CO_2_ into organic carbon through photosynthesis, thereby effectively reducing atmospheric CO_2_ concentrations and achieving sustained carbon fixation and carbon sink enhancement (Zhao et al., 2023). Leaves are the primary organs responsible for photosynthesis and carbon fixation. Investigating photosynthetic carbon fixation at the leaf scale is fundamental to understanding plant carbon fixation efficiency (Qian J. et al., 2025; Xu M. et al., 2025). Effective photosynthetic carbon fixation of plants depends on their successful adaptation and healthy growth within local environments. Leaves are among the most sensitive and plastic plant organs in their response to changing environmental conditions (Zhang et al., 2020). Consequently, the ability of leaf functional traits to reflect plant adaptation strategies and resource allocation patterns has been widely recognized (Li and Prentice, 2024; Wu et al., 2023). Complex intrinsic relationships exist among the structural, chemical, and physiological traits of leaves across species. Wright et al. (2004) proposed the leaf economics spectrum (LES), which for the first time quantitatively described the synergies and trade-offs among leaf functional traits at a global scale, and quantitatively characterized plant resource-use strategies. Specifically, plants with high specific leaf area, high leaf nitrogen content, high photosynthetic rates, and short leaf lifespans tend to adopt an acquisitive strategy, whereas those with high leaf mass per area, low nitrogen content, low photosynthetic rates, and long leaf lifespans typically exhibit a conservative strategy (Osnas et al., 2013; Wright et al., 2004). Since its proposal, the LES has garnered widespread attention in academia and has been applied across numerous research fields (Dong et al., 2020; Kunstler et al., 2016; Xu H. et al., 2025).

Previous studies have mainly focused on the responses of plant leaf functional traits and the LES to environmental changes (Yan et al., 2025; Zhu et al., 2023). As research has progressed, the indicative role of leaf functional traits in ecosystem services has received increasing attention (Hanisch et al., 2020; Pan et al., 2022; Zhang and Liu, 2020), but relevant studies remain relatively scarce. Leaf functional traits are linked to their ecological functions through physiological and biochemical processes, providing an important perspective for understanding the mechanisms of plant ecological functions. However, the relationship between leaf functional traits and photosynthetic carbon fixation in urban green space plants, especially landscape shrubs, has been scarcely explored. Plants from different geographical origins, life forms, and ecotypes often exhibit marked differences in carbon fixation capacity (Qian J. et al., 2025; Xu M. et al., 2025). However, which key leaf functional traits can effectively indicate the carbon fixation capacity of plants remains unclear. In studies on plant carbon fixation, photosynthetic capacity has been the most commonly used indicator of carbon fixation capacity. Most existing studies have examined the relationship between leaf traits and plant photosynthesis at the micro-physiological level (Burnett et al., 2021; Xu M. et al., 2025). Leaf functional traits such as specific leaf area (SLA), leaf mass per area (LMA), leaf dry matter content (LDMC), nitrogen (N), chlorophyll (Chl), and photosynthetic nitrogen use efficiency (PNUE) are widely recognized as key factors influencing photosynthetic capacity (Dusenge et al., 2020; Fan et al., 2024; Kattge et al., 2009; Montpied et al., 2009; Qian X. et al., 2025). Nevertheless, the correlation between LMA and photosynthetic capacity has been inconsistent across studies (Chen et al., 2024; Garnier et al., 2025; Niinemets, 2001), indicating uncertainty in the relationship between leaf functional traits and photosynthetic capacity. Consequently, relying solely on photosynthetic capacity to assess plant carbon fixation may have limitations, making it difficult to accurately identify the leaf functional traits that confer high carbon fixation capacity. According to the LES, plants with shorter leaf lifespans generally exhibit higher instantaneous photosynthetic rates, indicating greater instantaneous carbon fixation capacity (Wright et al., 2004). However, annual carbon fixation per unit leaf area is accumulated through photosynthesis, and long-lived leaves provide a longer period for carbon accumulation. Whether the trade-offs among leaf functional traits influence the total carbon assimilation of plant leaves during the growing season remains unclear. Furthermore, although the general applicability of the LES has been well validated in natural ecosystems and certain plant groups (Henneron et al., 2020; Xiong and Flexas, 2018; Zhang et al., 2024), whether this theory can explain the carbon fixation strategies of landscape shrubs in highly managed urban environments remains underexplored. Therefore, elucidating the mechanisms of photosynthetic carbon fixation in landscape shrubs from the perspective of leaf functional traits, and identifying key traits indicative of high carbon fixation capacity, are of great scientific and practical value for selecting and breeding landscape shrubs with high carbon fixation capacity.

This study examined eight common landscape deciduous shrubs in Beijing, a representative warm-temperate city. We used two indicators of photosynthetic carbon fixation—maximum net photosynthetic rate per unit leaf area (P_max_) and annual carbon fixation per unit leaf area (YL)—to quantify carbon fixation at the leaf level. By measuring leaf functional traits and photosynthetic carbon fixation indicators, we analyzed the characteristics of these traits, their interrelationships, and their effects on photosynthetic carbon fixation. The study aimed to address the following questions: (1) What is the LES of these landscape deciduous shrubs? (2) How do leaf functional traits affect photosynthetic carbon fixation in these shrubs? (3) Which key leaf functional traits can serve as indicators of photosynthetic carbon fixation capacity? The findings are intended to provide a bioecological basis for selecting and breeding landscape shrubs with high carbon fixation capacity.

## 2 Materials and methods

### 2.1 Study site

This study was conducted in Haidian District, Beijing, a representative warm-temperate urban area situated at mid-latitudes (39°53′ to 40°09′ N, 116°03′ to 116°23′ E). The region experiences a typical warm-temperate, semi-humid to semi-arid monsoon climate characterized by hot, rainy summers with prevailing southeasterly winds, and cold, dry winters dominated by northwesterly winds, with relatively brief spring and autumn seasons. The mean annual temperature is 12.5 °C, with the coldest month (January) averaging -4.4 °C and the hottest month (July) averaging 26.5 °C. Annual rainfall averages 628.9 mm, falling mainly in summer (June to August). The study site is Dongsheng Bajia Country Park, which opened in 2009 and covers a total area of 81.96 hectares. The study area has relatively uniform soil, moisture, and temperature conditions, moderate plant maintenance, and generally flat terrain, representing a typical urban green space in Beijing.

### 2.2 Study species

Based on field surveys and literature review of urban green spaces in Beijing, eight common landscape deciduous shrubs were selected as experimental materials for their good adaptability, widespread use, and strong representativeness (**Table 1**). These species cover different plant growth forms and branch-leaf characteristics (e.g., leaf size, leaf shape, leaf thickness, branch and leaf density). All selected individuals were located in open areas within park green spaces, away from water bodies and buildings, with similar habitat conditions and free from shading by surrounding structures. For each species, five standard individuals were selected that were healthy, vigorous, unpruned, and free from pests or diseases. We included adult plants of different ages (5–15 years) to eliminate the effect of age variation on leaf functional traits.

**Table 1:** Basic information of the tested shrub species.

Shrub species	Abbreviation	Plant life form	Average plant height (m)	Average plant crown width (m)
*Hibiscus syriacus*	*H. syriacus*	deciduous shrub	3.20 ± 0.16	1.87 ± 0.25
*Rosa hybrida*	*R. hybrida*	deciduous shrub	1.02 ± 0.33	0.60 ± 0.09
*Sorbaria sorbifolia*	*S. sorbifolia*	deciduous shrub	1.20 ± 0.15	1.05 ± 0.09
*Deutzia parviflora*	*D. parviflora*	deciduous shrub	2.53 ± 0.21	2.93 ± 0.12
*Cornus alba*	*C. alba*	deciduous shrub	1.46 ± 0.37	2.00 ± 0.44
*Viburnum opulus* subsp. *calvescens*	*V. opulus*	deciduous shrub	2.65 ± 0.05	2.31 ± 0.50
*Lonicera maackii*	*L. maackii*	deciduous shrub	4.17 ± 0.62	3.88 ± 0.73
*Weigela florida*	*W. florida*	deciduous shrub	1.55 ± 0.11	1.84 ± 0.20

### 2.3 Measurement of leaf functional traits

#### 2.3.1 Leaf structural traits

The experiments were conducted in August 2023, during the peak growth period of the plants, to ensure that the measured leaf functional traits accurately reflected their physiological and ecological characteristics. From each standard individual, 16 fresh, fully expanded, healthy, and undamaged mature leaves were collected from the outer canopy at mid−height in the eastern, southern, western, and northern directions. Leaf area (LA, m^2^) was measured using a portable leaf area scanner (FS−leaf1000, China). Leaf thickness (LT, mm) was measured at four evenly distributed points on each leaf, avoiding the main vein, with a vernier caliper (0.02 mm). Fresh weight (FW, g) was recorded immediately after collection with an analytical balance (0.0001 g). Leaves were then soaked in darkness for 12 h, after which surface moisture was blotted with filter paper and saturated fresh weight (SFW, g) was recorded. Finally, leaves were placed in an oven at 105 °C for 1 h to deactivate enzymes, then dried at 75 °C to constant weight to obtain leaf dry weight (DW, g). Based on these measurements, the following derived traits were calculated using Equations 1–4: SLA (m^2^/g), LMA (g/m^2^), LDMC (g/g), and leaf relative water content (LRWC, %).

(1) SLA = (LA/DW)

(2) LMA = (DW/LA)

(3) LDMC = (DW/SFW)

(4) LRWC = [(FW−DW)/(SFW−DW)]×100%

#### 2.3.2 Leaf chemical traits

The dried leaves were finely ground using a high-throughput tissue grinder (Hannuo-48, China). Leaf nitrogen content per unit mass (N_mass_, g/100 g) was determined using the Kjeldahl method, and total phosphorus content per unit mass (P_mass_, g/100 g) was determined using the Mo-Sb colorimetric method. These data were used to calculate nitrogen content per unit leaf area (N_area_, g/m^2^) with Equation 5, and phosphorus content per unit leaf area (P_area_, g/m^2^) with Equation 6.

(5) $${\text{N}}_{\text{area}}{\text{ = N}}_{\text{mass}}\times 100/\text{SLA}$$

(6) $${\text{ P}}_{\text{area}}{\text{ = P}}_{\text{mass}}\times 100/\text{SLA}$$

#### 2.3.3 Leaf physiological traits

Field experiments were conducted under clear, windless conditions during the summer (June to August) of 2023. Under natural light from 9:00 to 11:00, three to five intact, healthy, and fully expanded leaves were selected from the outer canopy of each tested species. Physiological parameters, including P_max_ (μmol·m^-2^·s^-1^), maximum transpiration rate per unit leaf area (Tr, mmol·m^-2^·s^-1^), and stomatal conductance (Gs, mol·m^-2^·s^-1^), were measured using the Li-6400XT portable photosynthesis system (LI−COR, USA). During measurements, leaves were flattened inside the standard leaf chamber without overlapping or shading each other. After readings stabilized, five measurements were taken per leaf and averaged. Concurrently, the relative chlorophyll content (SPAD value) of the leaves was measured with a portable chlorophyll meter (SPAD−502, Japan). Prior to measurements, the instrument was calibrated. Measurements were taken at three different locations on each leaf, avoiding the main vein, and the average was recorded as the representative SPAD reading (Marenco et al., 2009). PNUE (μmol·g^-1^·s^-1^) was shown in Equation 7.

(7) $${\text{PNUE = P}}_{\text{max}}/{\text{N}}_{\text{area}}$$

#### 2.3.4 Leaf lifespan

In this study, leaf lifespan was estimated as the average length of the growing season. The growing season lengths of the studied shrubs were obtained from relevant studies on the phenology of woody plants in Beijing and from the *Phenology Manual of Woody Plants in Beijing Urban Green Spaces* (Dong, 2020; Xing et al., 2018), and these values were used as leaf lifespan for each species.

### 2.4 Quantification of photosynthetic carbon fixation

#### 2.4.1 Measurement of leaf net photosynthetic rate

Photosynthetic rate measurements were conducted in the spring (March to May), summer (June to August), and autumn (September to November) of 2023, covering the complete growing season of deciduous shrubs from leaf expansion to leaf fall. On each sampling day, clear and windless days were selected, and measurements were carried out under natural light to ensure that the conditions closely approximated the actual growth environment of the plants. The instantaneous photosynthetic rates were measured every two hours from 7:00 to 17:00 using the Li-6400XT portable photosynthesis system (LI−COR, USA). For each species, three to five intact, healthy, and fully expanded leaves were selected from the outer canopy. The leaves were flattened inside the standard leaf chamber without overlapping. After readings stabilized, five data points were recorded per leaf and averaged. The net photosynthetic rate (p_n_, μmol·m^-2^·s^-1^) for each time interval was recorded, along with the maximum daily net photosynthetic rate observed over the daily measurement period (P_max_, μmol·m^-2^·s^-1^).

#### 2.4.2 Calculation of photosynthetic carbon fixation per leaf area

The daily net assimilation per unit leaf area was calculated using Equation 8.

(8) $$\text{P}\;=\;\sum_{\text{i}=1}^{\text{j}}\;[\frac{{\text{p}}_{\text{n},\text{i}}+{\text{p}}_{\text{n},\text{i}+1}}{2}\times ({\text{t}}_{\text{i}+1}-{\text{t}}_{\text{i}})\times \frac{3600}{1000}]$$

where P is the net daily carbon assimilation per unit leaf area on the measurement day (mmol·m^-2^·d^-1^); P_n,i_ and P_n,i+1_ are the instantaneous photosynthetic rates (μmol·m^-2^·s^-1^) at the initial and next measurement time points; t_i_ and t_i+1_ are the corresponding time points (h); j is the total number of measurements; 3600 is the number of seconds per hour; and 1000 converts μmol to mmol.

The daily carbon fixation per unit leaf area was then derived using Equation 9.

(9) W = P×(1−20%)×44/1000

where W is the daily carbon fixation per unit leaf area (g·m^-2^·d^-1^); nighttime dark respiration in plants generally consumes approximately 20% of the carbon assimilated during the day (Jin et al., 2023); and 44 is the molar mass of CO_2_.

The annual carbon fixation per unit leaf area was estimated by Equation 10 as follows:

(10) $$\text{YL = }({\text{W}}_{\text{c}}+{\text{W}}_{\text{x}}+{\text{W}}_{\text{q}})/3\times \text{LL}$$

where YL is the annual carbon fixation per unit leaf area for deciduous shrubs (g·m^-2^·a^-1^); W_c_, W_x_, W_q_ are the daily carbon fixation per unit leaf area (g·m^-2^·d^-1^) measured in spring, summer, and autumn, respectively; and LL is the average length of the growing season for deciduous shrubs (d) in the Beijing region (Xing et al., 2018).

### 2.5 Data analysis

Mean values and standard deviations were first calculated for all measured leaf functional traits and photosynthetic carbon fixation indicators of the landscape deciduous shrubs. Data were then tested for normality and homogeneity of variance using the Shapiro-Wilk test and Levene’s test, respectively. Significant differences in these traits and indicators among shrub species were assessed using one-way analysis of variance (ANOVA), followed by Duncan’s multiple range test for *post-hoc* comparisons. Pearson correlation analysis was used to examine the correlations among leaf functional traits, as well as between these traits and photosynthetic carbon fixation indicators. We used principal component analysis (PCA) to ordinate the 12 leaf functional traits of the shrubs. Based on these results, we analyzed their position along the LES and explored the similarities and differences in resource-use strategies among species. Subsequently, the effects of leaf functional traits on photosynthetic carbon fixation were evaluated using partial least squares (PLS) regression. The relative importance of each predictor was quantified by the variable importance in projection (VIP) score. Predictors with VIP > 1 were considered to contribute significantly to the model. All statistical computations and analyses were performed using Excel 2016, SPSS 27.0, and SIMCA 14.1. All figures were generated using Origin 2024.

## 3 Results

### 3.1 The leaf economics spectrum in eight deciduous shrub species

#### 3.1.1 Leaf functional traits of shrubs

**Figure 1:**
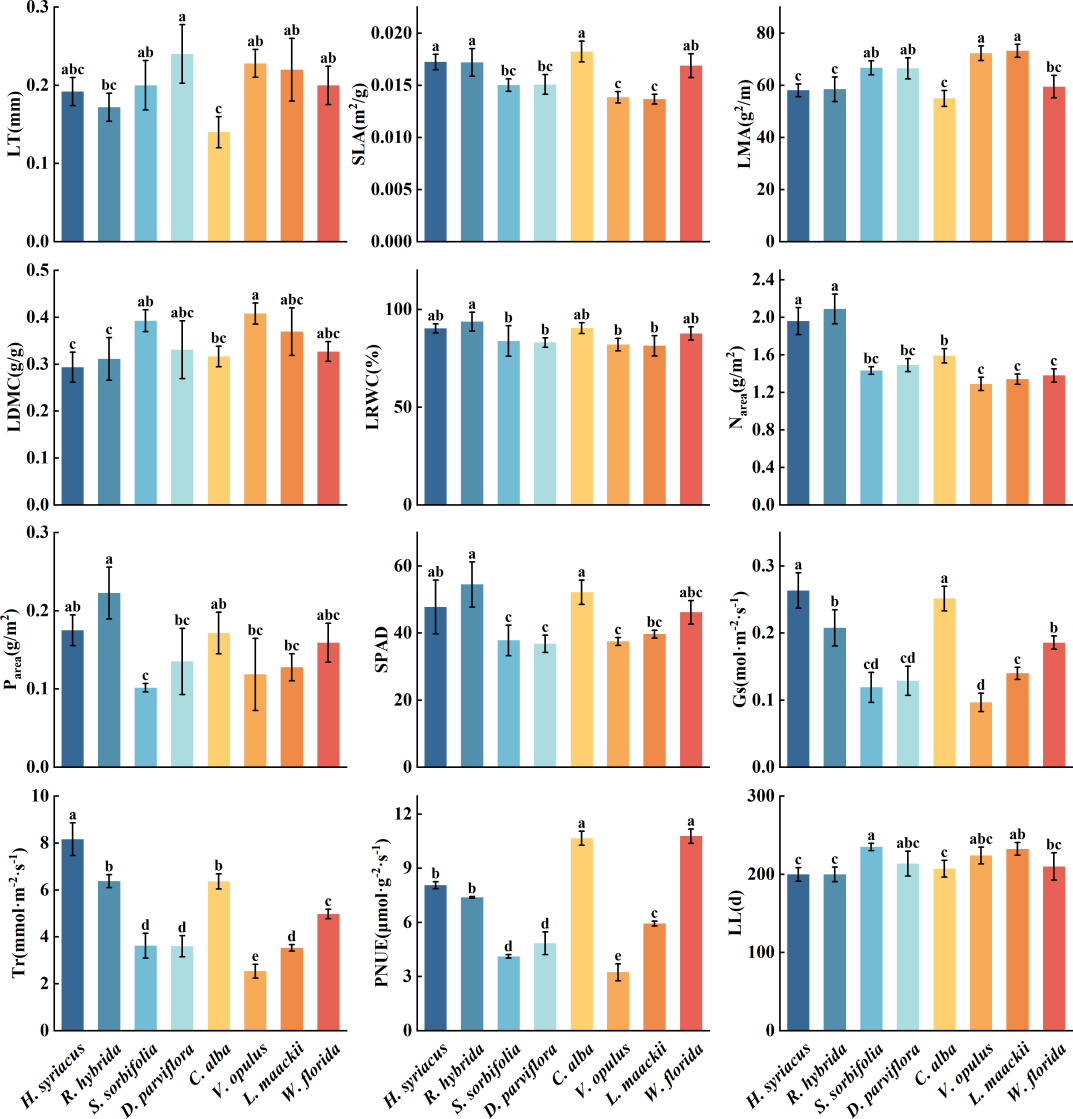
Leaf functional traits of eight landscape deciduous shrubs. LT: leaf thickness (mm); SLA: specific leaf area (m^2^/g); LMA: leaf mass per area (g/m^2^); LDMC: leaf dry matter content (g/g); LRWC: leaf relative water content (%); N_area_: nitrogen content per unit leaf area (g/m^2^); P_area_: phosphorus content per unit leaf area (g/m^2^); SPAD: relative chlorophyll content; Gs: stomatal conductance (mol·m^-2^·s^-1^); Tr: maximum transpiration rate per unit leaf area (mmol·m^-2^·s^-1^); PNUE: photosynthetic nitrogen use efficiency (μmol·g^-1^·s^-1^); LL: leaf lifespan (d). Species are denoted by their initial-letter abbreviations on the x-axis. *H. syriacus*: *Hibiscus syriacus*; *R. hybrida*: *Rosa hybrida*; *S. sorbifolia*: *Sorbaria sorbifolia*; *D. parviflora*: *Deutzia parviflora*; *C. alba*: *Cornus alba*; *V. opulus*: *Viburnum opulus* subsp. *calvescens*; *L. maackii*: *Lonicera maackii*; *W. florida*: *Weigela florida*. The sample size for each shrub species was 5 (n = 5). Different lowercase letters above the bars indicate statistically significant differences among shrub species (*P* < 0.05).

As shown in **Figure 1**, leaf functional traits differed significantly among the eight shrub species (*P* < 0.05). Regarding leaf structural traits, the mean LT across species was 0.20 mm, with *D. parviflora* showing the highest value (0.24 mm) and *C. alba* the lowest (0.14 mm). SLA averaged 0.0159 m^2^/g, reaching a maximum of 0.0182 m^2^/g in *C. alba* and a minimum of 0.0137 m^2^/g in *L. maackii*. LMA averaged 63.72 g/m^2^, with the maximum observed in *L. maackii* (73.13 g/m^2^) and the minimum in *C. alba* (54.83 g/m^2^). Mean LDMC was 0.34 g/g, peaking in *V. opulus* (0.41 g/g) and reaching its lowest level in *H. syriacus* (0.29 g/g). LRWC averaged 86.14%, with the highest value recorded for *R. hybrida* (93.74%) and the lowest for *L. maackii* (81.40%).

For leaf chemical traits, the mean N_area_ across the eight shrub species was 1.59 g/m^2^. The N_area_ of *R. hybrida* and *H. syriacus* was significantly higher than that of the other species (*P* < 0.05), with values of 2.09 g/m^2^ and 1.96 g/m^2^, respectively. In contrast, *V. opulus* exhibited the lowest N_area_ (1.29 g/m^2^). The mean P_area_ was 0.15 g/m^2^, with the highest value recorded in *R. hybrida* (0.22 g/m^2^) and the lowest in *S. sorbifolia* (0.10 g/m^2^).

Regarding leaf physiological traits, the measured traits varied among the eight species as follows. The mean SPAD was 44.07, with the highest value recorded in *R. hybrida* (54.50) and the lowest in *S. sorbifolia* (36.80). Gs averaged 0.17 mol·m^-2^·s^-1^; *H. syriacus* and *C. alba* exhibited significantly higher Gs values than the other species (*P* < 0.05), reaching maxima of 0.26 mol·m^-2^·s^-1^ and 0.25 mol·m^-2^·s^-1^, respectively, while the minimum was found in *V. opulus* (0.10 mol·m^-2^·s^-1^). The mean maximum Tr was 4.90 mmol·m^-2^·s^-1^. Tr was significantly higher in *H. syriacus* (8.16 mmol·m^-2^·s^-1^; *P* < 0.05) than in the other species, and lowest in *V. opulus* (2.54 mmol·m^-2^·s^-1^). For PNUE, the values for *W. florida* and *C. alba* were significantly higher than those for the remaining species (*P* < 0.05), at 10.78 μmol·g^-1^·s^-1^ and 10.67 μmol·g^-1^·s^-1^, respectively. In contrast, *S. sorbifolia* exhibited the lowest PNUE (4.12 μmol·g^-1^·s^-1^).

#### 3.1.2 Trade-offs and synergies among leaf functional traits in shrubs

**Figure 2:**
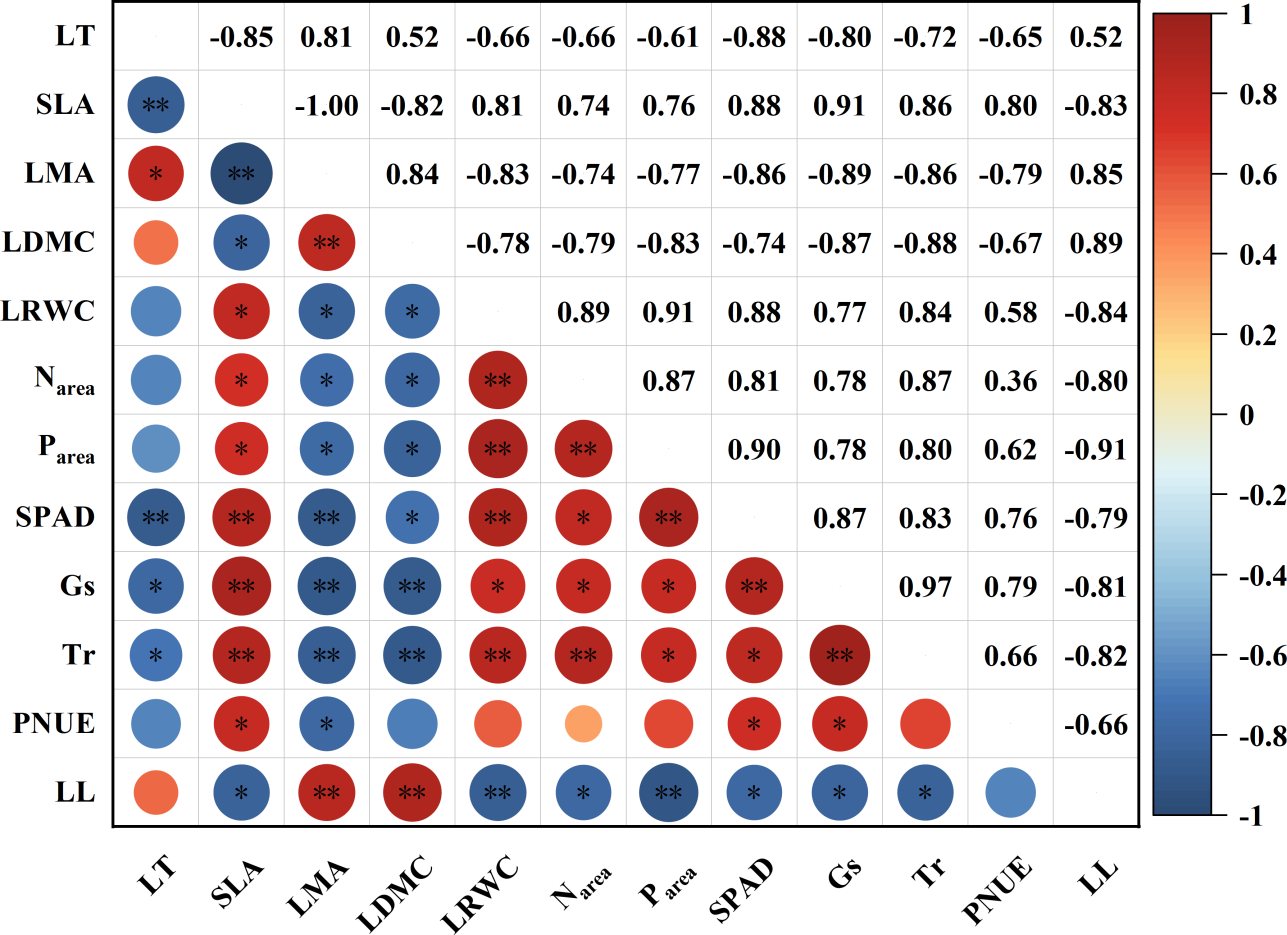
Correlations among leaf functional traits in eight landscape deciduous shrubs. LT: leaf thickness (mm); SLA: specific leaf area (m^2^/g); LMA: leaf mass per area (g/m^2^); LDMC: leaf dry matter content (g/g); LRWC: leaf relative water content (%); N_area_: nitrogen content per unit leaf area (g/m^2^); P_area_: phosphorus content per unit leaf area (g/m^2^); SPAD: relative chlorophyll content; Gs: stomatal conductance (mol·m^-2^·s^-1^); Tr: maximum transpiration rate per unit leaf area (mmol·m^-2^·s^-1^); PNUE: photosynthetic nitrogen use efficiency (μmol·g^-1^·s^-1^); LL: leaf lifespan (d). Asterisks denote statistical significance: *P < 0.05, **P < 0.01.

Significant correlations were observed among multiple leaf functional trait groups of the eight shrub species in Beijing (**Figure 2**). Among leaf structural traits, SLA exhibited a highly significant negative correlation with LT and LMA (*P* < 0.01), and a significant negative correlation with LDMC (*P* < 0.05). Conversely, LMA showed a highly significant positive correlation with LDMC (*P* < 0.01) and a significant positive correlation with LT (*P* < 0.05). Regarding leaf chemical traits, N_area_ showed a highly significant positive correlation with P_area_ (*P* < 0.01). For leaf physiological traits, Gs was strongly positively correlated with Tr (*P* < 0.01). PNUE exhibited significant positive correlations with SPAD and with Gs (*P* < 0.05).

Close associations were identified between leaf chemical traits and structural traits. Both N_area_ and P_area_ exhibited highly significant positive correlations with LRWC (*P* < 0.01) and significant positive correlations with SLA (*P* < 0.05). In contrast, they were significantly negatively correlated with LMA and LDMC (*P* < 0.05). Significant correlations were also evident between physiological traits and structural traits. Specifically, SPAD exhibited highly significant positive correlations with SLA and LRWC (*P* < 0.01), and highly significant negative correlations with LT and LMA (*P* < 0.01). Its correlation with LDMC was significantly negative (*P* < 0.05). PNUE was positively correlated with SLA and negatively correlated with LMA, both at the *P* < 0.05 significance level. Regarding the relationships between physiological and chemical traits, SPAD, Gs, and Tr all showed positive correlations ranging from significant to highly significant with both N_area_ and P_area_. Furthermore, LL displayed highly significant positive correlations with LMA and LDMC (*P* < 0.01). Conversely, it was highly significantly negatively correlated with LRWC and P_area_ (*P* < 0.01), and significantly negatively correlated with SLA, N_area_, SPAD, Gs, and Tr (*P* < 0.05). The network of significant positive correlations among SLA, LRWC, N_area_, P_area_, SPAD, Gs, Tr, and PNUE indicates that these traits covary synergistically. Conversely, their significant negative correlations with LMA, LDMC, and LL suggest a trade-off relationship, reflecting contrasting resource investment strategies.

**Figure 3:**
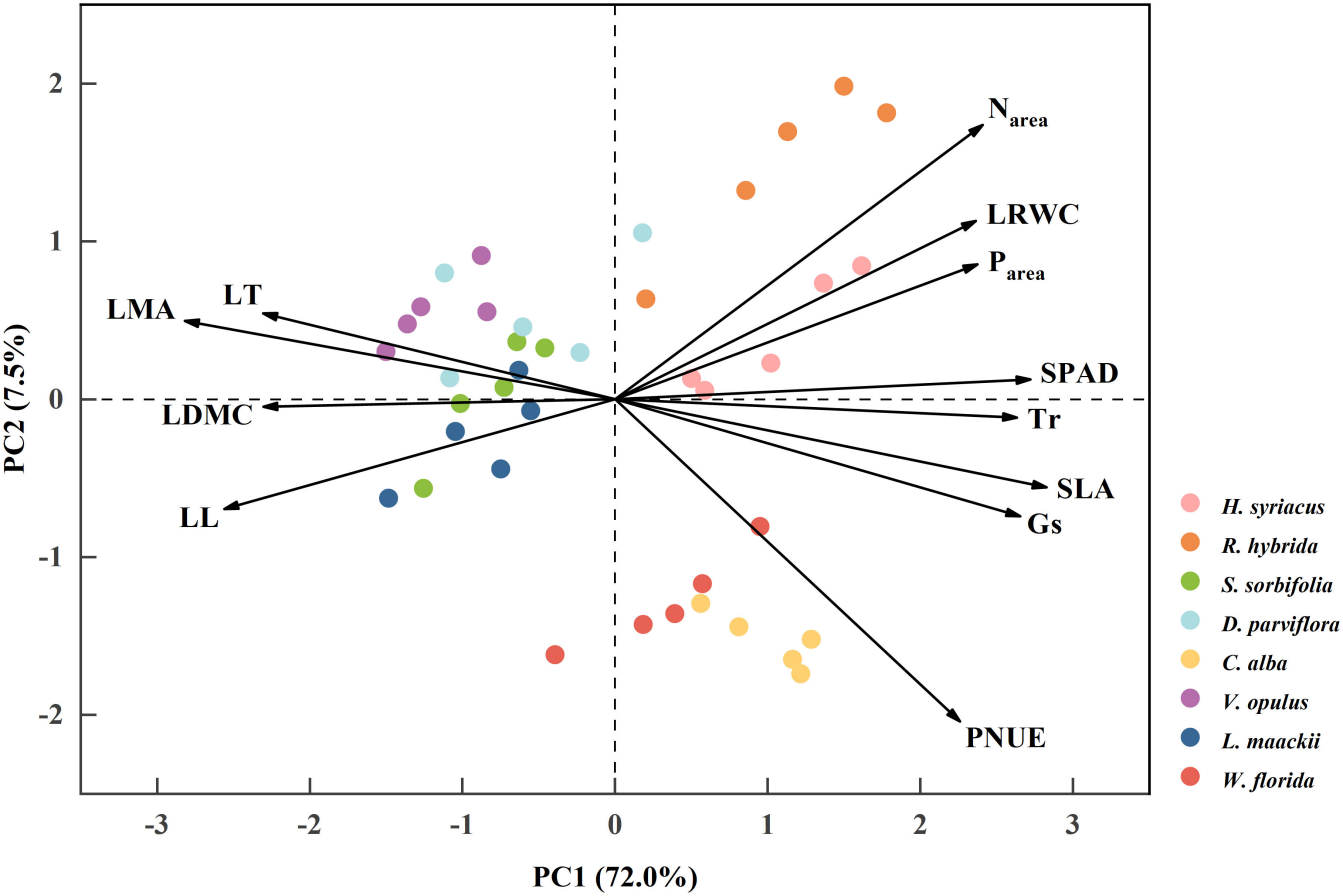
Principal component analysis (PCA) of leaf functional traits in eight landscape deciduous shrubs. LT: leaf thickness (mm); SLA: specific leaf area (m^2^/g); LMA: leaf mass per area (g/m^2^); LDMC: leaf dry matter content (g/g); LRWC: leaf relative water content (%); N_area_: nitrogen content per unit leaf area (g/m^2^); P_area_: phosphorus content per unit leaf area (g/m^2^); SPAD: relative chlorophyll content; Gs: stomatal conductance (mol·m^-2^·s^-1^); Tr: maximum transpiration rate per unit leaf area (mmol·m^-2^·s^-1^); PNUE: photosynthetic nitrogen use efficiency (μmol·g^-1^·s^-1^); LL: leaf lifespan (d). Species abbreviations are used on the x-axis. *H. syriacus*: *Hibiscus syriacus*; *R. hybrida*: *Rosa hybrida*; *S. sorbifolia*: *Sorbaria sorbifolia*; *D. parviflora*: *Deutzia parviflora*; *C. alba*: *Cornus alba*; *V. opulus*: *Viburnum opulus* subsp. *calvescens*; *L. maackii*: *Lonicera maackii*; *W. florida*: *Weigela florida*.

PCA results (**Figure 3**) showed that the first principal component (PC1) and the second principal component (PC2) explained 72.0% and 7.5% of the total variance, respectively. Together, the first two principal components accounted for 79.5% of the total variance in the original 12 leaf functional traits, retaining most of the original information and effectively capturing the relationships among the traits, with PC1 largely determining the overall structure. PC1 was strongly correlated with key leaf functional traits, including SLA, SPAD, LMA, and LL, and can be interpreted as the resource strategy axis of the LES, reflecting a continuous gradient from conservative to acquisitive strategies. Among the studied species, *H. syriacus*, *R. hybrida*, *C. alba*, and *W. florida* were mainly distributed on the positive side of the PC1 axis. These species exhibited higher SLA, SPAD, Gs, Tr, N_area_, P_area_, LRWC, and PNUE, indicating an acquisitive strategy. In contrast, *S. sorbifolia*, *D. parviflora*, *V. opulus*, and *L. maackii* were mainly distributed on the negative side of the PC1 axis, showing higher LMA, LL, LT, and LDMC, representing the conservative strategy at the opposite end of the LES.

### 3.2 Maximum net photosynthetic rate and annual carbon assimilation in eight deciduous shrubs

Two key indicators, P_max_ and YL, were used to assess photosynthetic carbon fixation capacity. The former represents the instantaneous carbon fixation potential of leaf tissue, while the latter, derived from net photosynthetic rates, reflects carbon assimilation capacity on an annual scale.

**Figure 4:**
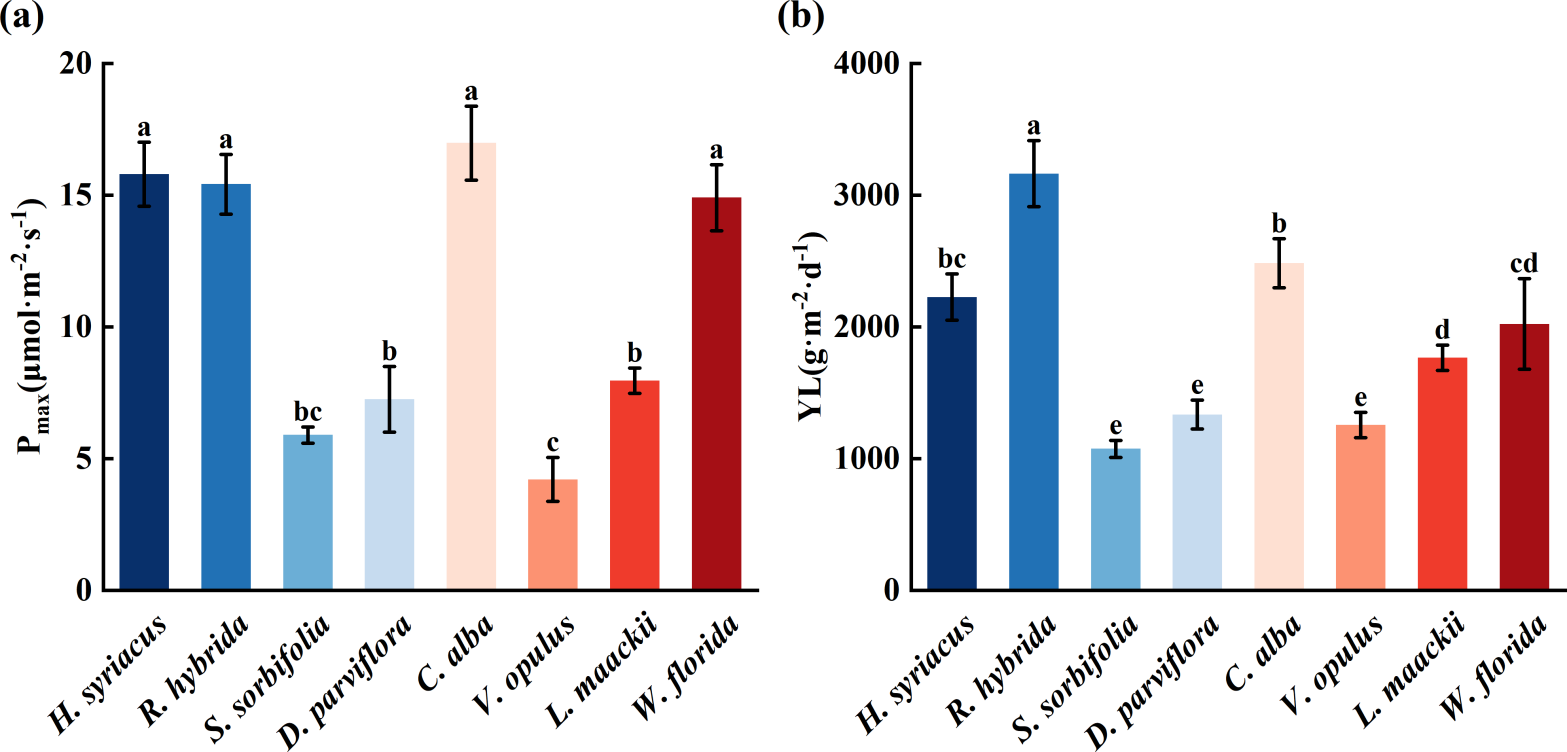
**(a)** Maximum net photosynthetic rate per unit leaf area (P_max_, μmol·m^-2^·s^-1^) of eight landscape deciduous shrubs. **(b)** Annual carbon fixation per unit leaf area (YL, g·m^-2^·a^-1^) of eight species. Species are denoted by their abbreviations on the x-axis. *H. syriacus*: *Hibiscus syriacus*; *R. hybrida*: *Rosa hybrida*; *S. sorbifolia*: *Sorbaria sorbifolia*; *D. parviflora*: *Deutzia parviflora*; *C. alba*: *Cornus alba*; *V. opulus*: *Viburnum opulus* subsp. *calvescens*; *L. maackii*: *Lonicera maackii*; *W. florida*: *Weigela florida*. The sample size for each shrub species was 5 (n = 5). Different lowercase letters above the bars indicate statistically significant differences among shrub species (*P* < 0.05).

P_max_ among the eight species ranged from 5.79 to 16.98 μmol·m^-2^·s^-1^, with a mean value of 11.26 μmol·m^-2^·s^-1^. C*. alba* exhibited the highest P_max_ (16.98 μmol·m^-2^·s^-1^), followed by *H. syriacus* (15.80 μmol·m^-2^·s^-1^) and *R. hybrida* (15.42 μmol·m^-2^·s^-1^). In contrast, *V. opulus* and *S. sorbifolia* had the lowest values, at 5.79 and 5.90 μmol·m^-2^·s^-1^, respectively (**Figure 4A**).

YL ranged from 1073.68 to 3162.83 g·m^-2^·a^-1^, with a mean of 1915.35 g·m^-2^·a^-1^. Specifically, *R. hybrida* exhibited significantly higher annual carbon fixation (3162.83 g·m^-2^·a^-1^) than all other species. *C. alba* and *H. syriacus* ranked second and third, with values of 2482.56 and 2226.10 g·m^-2^·a^-1^, respectively. In contrast, *S. sorbifolia* (1073.68 g·m^-2^·a^-1^), *V. opulus* (1255.87 g·m^-2^·a^-1^), and *D. parviflora* (1334.00 g·m^-2^·a^-1^) showed significantly lower annual carbon fixation (**Figure 4B**).

### 3.3 Linking leaf functional traits to photosynthetic carbon fixation in eight deciduous shrubs

Significant correlations were identified between leaf functional traits and photosynthetic carbon fixation indicators across the eight species (**Table 2**). Specifically, P_max_ exhibited highly significant positive correlations (*P* < 0.01) with SLA, P_area_, SPAD, and Gs. It also showed significant positive correlations (*P* < 0.05) with N_area_, Tr, and PNUE. Conversely, P_max_ was highly significantly negatively correlated (*P* < 0.01) with LMA and LDMC, and significantly negatively correlated (*P* < 0.05) with LT and LL.

**Table 2:** Correlation analysis between leaf functional traits and photosynthetic carbon fixation indicators.

Leaf functional traits	P_max_	YL
LT	-0.826*	-0.766*
SLA	0.857**	0.762*
LMA	-0.850**	-0.790*
LDMC	-0.886**	-0.862**
LRWC	0.667	0.714*
N_area_	0.714*	0.714*
P_area_	0.857**	0.952**
SPAD	0.857**	0.905**
Gs	0.976**	0.881**
Tr	0.833*	0.762*
PNUE	0.810*	0.762*
LL	-0.802*	-0.886**

Asterisks denote statistical significance: *P < 0.05, **P < 0.01.

YL also displayed significant relationships with multiple leaf functional traits. It showed highly significant positive correlations (*P* < 0.01) with P_area_, SPAD, and Gs. Additionally, it was significantly positively correlated (*P* < 0.05) with SLA, LRWC, N_area_, Tr, and PNUE. Conversely, YL was highly significantly negatively correlated (*P* < 0.01) with LDMC and LL, and significantly negatively correlated (*P* < 0.05) with LT and LMA.

These correlations suggest that the suite of acquisitive traits—including SLA, N_area_, P_area_, SPAD, Gs, Tr, and PNUE—likely functions synergistically to enhance photosynthetic carbon fixation capacity. Conversely, negative correlations were observed for conservative traits such as LT, LMA, LDMC, and LL. This indicates a fundamental trade-off in leaf economics, where investment in structural persistence compromises photosynthetic carbon gain.

### 3.4 Relative importance of leaf functional traits in explaining photosynthetic carbon fixation

**Figure 5:**
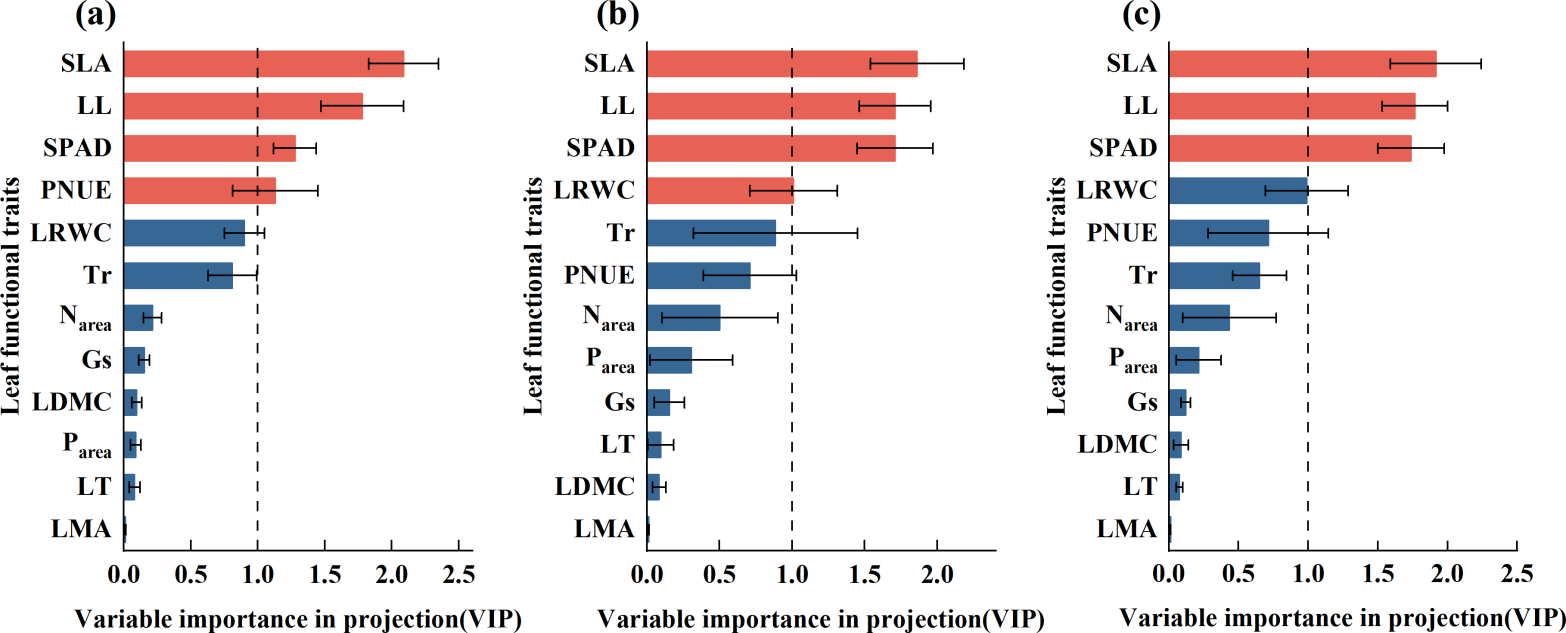
Effects of leaf functional traits on photosynthetic carbon fixation based on partial least squares (PLS) regression analysis (mean ± SE). **(a)** Variable importance in projection (VIP) of leaf functional traits in explaining P_max_. **(b)** VIP of traits in explaining YL. **(c)** VIP of traits in explaining photosynthetic carbon fixation. LT: leaf thickness (mm); SLA: specific leaf area (m^2^/g); LMA: leaf mass per area (g/m^2^); LDMC: leaf dry matter content (g/g); LRWC: leaf relative water content (%); N_area_: nitrogen content per unit leaf area (g/m^2^); P_area_: phosphorus content per unit leaf area (g/m^2^); SPAD: relative chlorophyll content; Gs: stomatal conductance (mol·m^-2^·s^-1^); Tr: maximum transpiration rate per unit leaf area (mmol·m^-2^·s^-1^); PNUE: photosynthetic nitrogen use efficiency (μmol·g^-1^·s^-1^); LL: leaf lifespan (d). In PLS regression, traits with VIP > 1 are considered important contributors to the model. Red bars indicate leaf functional traits with VIP > 1 (contributing more to photosynthetic carbon fixation), and blue bars indicate those with VIP < 1 (contributing less).

Based on leaf functional traits, we constructed three PLS models to assess the importance of these traits for photosynthetic carbon fixation in eight species. The models targeted P_max_, YL, and the integrated photosynthetic carbon fixation derived from these two indicators, respectively (**Figure 5**). For the model elucidating P_max_, the goodness-of-fit parameters were high (R^2^ = 0.986, Q^2^ = 0.978), both approaching 1, indicating strong explanatory and predictive power. The YL model also performed well (R^2^ = 0.931, Q^2^ = 0.802), with all parameters exceeding the 0.5 threshold, confirming its stability and reliability. The combined model (all traits as independent variables; Pmax and YL as dependent variables) yielded satisfactory parameters (R^2^ = 0.872, Q^2^ = 0.716), also above 0.5, indicating an acceptable overall fit.

The Variable Importance in Projection (VIP) statistic from the PLS models was used to quantify the contribution of each leaf functional trait to explaining variance in the response variables. A VIP value greater than 1 is generally considered indicative of an important predictor. For the model explaining P_max_, the traits with VIP > 1 were SLA, LL, SPAD, and PNUE (**Figure 5A**). These traits were identified as key drivers of instantaneous carbon fixation potential. In the YL model, the important traits (VIP > 1) were SLA, LL, SPAD, and LRWC (**Figure 5B**), highlighting their role as primary determinants of cumulative annual carbon gain. Examining the overall pattern across the models, SLA, LL, and SPAD consistently emerged as influential factors for photosynthetic carbon fixation in general (**Figure 5C**), with SLA exhibiting the strongest effect.

## 4 Discussion

### 4.1 Adaptation strategies of deciduous shrubs based on the leaf economics spectrum

Plant functional traits directly influence growth, development, survival, and reproduction, objectively reflecting their adaptive capacity to external environments (Duan et al., 2022; Lei et al., 2025). LRWC remained consistently high across all studied species. This favorable status can be attributed to the suitable climatic and edaphic conditions of the urban green spaces, which enabled the plants to sustain stable water relations. Under the same urban habitat, significant variation in leaf functional traits was observed among different shrubs. This indicates divergence in leaf structure, nutrient composition, and photosynthetic strategies among species, even under comparable growing conditions, reflecting the diversity of plant responses to the environment (Akram et al., 2022). This variation is closely linked to intrinsic factors, particularly genetic variation (Challis et al., 2024). Within the same climatic zone, plant leaf functional traits can also vary due to differences in environmental factors such as microclimate, topography, and elevation (Yuan et al., 2023). In this study, *H. syriacus*, *R. hybrida*, and *C. alba* generally exhibited higher SLA, LRWC, and Tr than did the same shrub specie in Lanzhou, China, while their LDMC was relatively lower (Li and Tian, 2022). This may be related to the semi-arid climatic conditions of Lanzhou. In a relatively arid urban environment, plants typically exhibit decreased leaf water content (Jin et al., 2015). They adapt by increasing LT, elevating LDMC, and reducing SLA (Wang S. et al., 2024; Wu et al., 2022). These adjustments help minimize water loss through transpiration and mitigate drought-induced leaf damage, thereby enhancing survival in semi-arid environments.

During growth and environmental adaptation, plants adjust functional traits of their organs under genetic, phylogenetic, and environmental influences to form an optimal combination for specific conditions (Xiong et al., 2022). This reflects distinct ecological strategies in response to their habitat. The eight shrub species exhibited clear divergence in leaf traits, forming two typical resource-use strategies. *H. syriacus*, *R. hybrida*, *C. alba*, and *W. florida* collectively displayed higher SLA, LRWC, N_area_, P_area_, SPAD, Gs, Tr, and PNUE, along with lower LT, LMA, LDMC, and shorter LL. This pattern aligns with previous findings (Wang N. et al., 2024; Zhang et al., 2025). This suite of traits reflects an acquisitive strategy, whereby carbon, N, and other resources are rapidly allocated to photosynthetic tissues under favorable conditions to enhance light capture efficiency and support rapid growth (Yan et al., 2025). In contrast, *S. sorbifolia*, *D. parviflora*, *V. opulus*, and *L. maackii* exhibited the opposite set of traits, indicating a more conservative strategy. This strategy prioritizes allocating limited resources to cell-wall thickening, structural biomass accumulation (Lichstein et al., 2021), and defense compound synthesis, thereby enhancing survival under stressful or resource-limited conditions (Hao et al., 2025).

### 4.2 Synergistic and trade-off relationships among leaf functional traits in deciduous shrubs

Plant adaptation to the environment does not rely on changes in single traits but involves coordinated resource allocation through synergies and trade-offs among multiple leaf functional traits (Wright et al., 2007). This leads to intrinsic correlations. Zhang et al. (2025) studied subtropical urban tree species. They found that the differences in key leaf functional traits related to wood properties and leaf habit, as well as their synergistic and trade-off relationships, follow the LES. In this study, the leaf functional traits of the eight species were highly correlated. This is generally consistent with global correlations among plant leaf traits (Wright et al., 2004). This reflects the convergence of plant adaptation strategies to the environment and confirms the applicability of the LES in warm-temperate urban habitats.

Among the eight deciduous shrubs, a significant negative correlation was observed between SLA (reflecting light acquisition capacity) and LDMC (representing nutrient retention capacity). This aligns with the resource allocation hypothesis, which posits that increased investment in leaf physical defense corresponds to reduced investment in photosynthetic capacity (Díaz et al., 2016). This further indicates a trade-off between growth and enhanced defense in plants (Dai et al., 2022). LMA showed significant positive correlations with both LDMC and LT, consistent with findings by Fan et al. (2024) and Shi et al. (2024). Plants with higher LMA typically exhibit greater LDMC and possess thicker, denser leaf structures. This enhances their capacity to retain nutrients and water, thereby improving resistance to environmental stress (Niinemets, 2001). Among leaf chemical traits, N_area_ and P_area_ showed a highly significant positive correlation. N and phosphorus are essential nutrients for plant growth and development, and they function in close coordination within physiological and metabolic processes (Wright et al., 2005). Significant correlations were also found among physiological traits. Photosynthetic parameters such as Gs and Tr reflect a plant’s capacity to utilize light energy. In this study, Tr and Gs exhibited a highly significant positive correlation, which is consistent with the results of Sheshshayee et al. (2005). Gs is the primary direct factor determining Tr, as stomata regulate their aperture via guard cells, thereby controlling water loss and influencing transpiration (Blatt et al., 2022).

Close linkages exist among leaf structural, chemical, physiological traits, as well as with leaf lifespan. In this study, SPAD was significantly positively correlated with N_area_, primarily because N is closely involved in Chl synthesis (Wen et al., 2019) and enhances photosynthetic efficiency by promoting both Chl production and cell expansion (Yao et al., 2018). N_area_ and P_area_ both showed significant correlations with structural traits such as SLA, LDMC, and LRWC, underscoring the critical role of nutrients in shaping plant structure (Wang et al., 2020). N_area_ correlated positively with SLA, and both traits were negatively correlated with LL. In contrast, LL, LMA, and LDMC showed highly significant positive correlations with each other. This pattern aligns with previous studies (Tsujii et al., 2023; Yang et al., 2019) and reflects a core “investment-return” trade-off in plant resource allocation. Higher SLA enables greater photosynthetic area per unit leaf mass, facilitating rapid light capture and carbon fixation (Liu et al., 2023). However, this typically requires substantial N investment to support a rich photosynthetic enzyme system (Nasar et al., 2022). Conversely, higher LMA and LDMC are often associated with thicker cell walls and a higher proportion of structural materials (Lu et al., 2020; Onoda et al., 2017). These traits enhance leaf resistance to mechanical damage, pests, diseases, and environmental stress, thereby extending leaf lifespan (Liu et al., 2022).

### 4.3 Effects of leaf functional traits on photosynthetic carbon fixation in deciduous shrubs

Plants adjust their functional traits to adapt to environmental changes during growth and development. However, optimal ecological performance depends on unimpeded plant growth and development. Moreover, plant functional traits can influence the expression of ecological effects (Shen et al., 2024; Wang et al., 2025). While adapting to the environment and maintaining healthy growth, different landscape tree species exhibit variations in photosynthetic carbon fixation capacity due to their distinct genetic characteristics. Notably, this study found that *C. alba*, *R. hybrida*, *H. syriacus*, and *W. florida* possess higher SLA, N_area_, P_area_, SPAD, Gs, Tr, and PNUE. These species also showed higher P_max_ and YL, demonstrating stronger photosynthetic carbon fixation capacity. In contrast, *S. sorbifolia*, *V. opulus*, *D. parviflora*, and *L. maackii* have higher LMA and LDMC, as well as longer LL. These species exhibited relatively lower P_max_ and YL, indicating weaker photosynthetic carbon fixation capacity. Li et al. (2023) also reported that, among four landscape deciduous shrubs in the Jianghuai region of China, *W. florida* and *C. alba*, which have larger LA, exhibited higher P_max_ and daily carbon fixation per unit leaf area. This study further revealed that leaf functional traits influence carbon fixation capacity by affecting leaf net photosynthetic rate and annual carbon assimilation.

P_max_ is a key indicator of plant photosynthetic capacity, reflecting the instantaneous maximum potential for carbon fixation. In this study, SLA, N_area_, and P_area_ all exerted positive effects on P_max_, which aligns with previous findings (Ni et al., 2024; Niinemets, 2023). High SLA implies that a larger leaf area is constructed per unit mass. This facilitates light capture and reduces CO_2_ transfer resistance in the mesophyll tissue, thereby promoting CO_2_ diffusion from the substomatal cavity to the chloroplasts (Liu et al., 2025). N is a key component of photosynthetic enzymes and chloroplasts. It regulates the activity of ribulose-1,5-bisphosphate carboxylase/oxygenase (Rubisco) and electron transport capacity, thereby influencing the maximum carboxylation rate and maximum electron transport rate (Woodrow and Berry, 1988). Phosphorus, as a key component of adenosine triphosphate (ATP), provides energy for carbon fixation. When sufficient, it drives ribulose-1,5-bisphosphate (RuBP) regeneration to maintain a high carboxylation rate (Sawada et al., 1992). Together, they influence photosynthetic carbon assimilation efficiency. Consequently, plants with high leaf N and phosphorus contents generally exhibit stronger photosynthetic capacity (Yong et al., 2025). P_max_ showed significant to highly significant positive correlations with physiological traits such as Gs, Tr, and SPAD. This is because stomata regulate plant gas exchange, thereby influencing both transpiration and carbon assimilation (Brodribb and Jordan, 2011; Ni et al., 2023). Higher Gs reduces the resistance to CO_2_ diffusion from the atmosphere to the carboxylation sites within mesophyll cells, thereby promoting photosynthetic carbon fixation (Wu et al., 2021). Chl is the primary photosynthetic pigment in the light-harvesting system, responsible for capturing light energy. Leaves with high P_max_ invest more resources in Chl synthesis to expand their light-capturing apparatus, thereby driving a more intensive carbon assimilation process (Zhang et al., 2019). We found that these three physiological traits are highly synergistic. This likely reflects a coordinated strategy where leaves ensure adequate CO_2_ supply by increasing stomatal aperture and secure energy supply by enhancing Chl content, together driving high P_max_. High transpiration rate is an inevitable consequence of this high-rate metabolic process. P_max_ was significantly negatively correlated with LT, LDMC, and LMA, consistent with related studies (Garnier et al., 2025; Wang and Jin, 2024). Higher LT and LDMC indicate greater investment in structural materials per unit area. Although this strategy helps prolong leaf lifespan, it reduces the proportion of leaf N allocated to photosynthetic proteins (Onoda et al., 2017). Meanwhile, cell wall thickening increases CO_2_ diffusion resistance within the mesophyll, leading to a decreased CO_2_ concentration at Rubisco and consequently reducing the photosynthetic rate (Garnier et al., 2025).

YL measures the total carbon assimilated by leaves over one year. The correlation patterns between YL and the leaf functional traits closely mirrored those between P_max_ and the same traits. For total annual leaf carbon gain, higher Gs and Tr represent a carbon acquisition trade-off strategy under favorable conditions, such as adequate water supply. Although this may reduce instantaneous water-use efficiency, it can enhance seasonal carbon gain by boosting carbon assimilation (Lawson and Matthews, 2020). SPAD exhibits a significant positive effect on YL. This is consistent with the findings of Jin et al. (2023) on daily carbon fixation per unit leaf area for tree and shrub species in urban green spaces in Zhengzhou, China. YL is also influenced by the duration of photosynthesis, which is determined by LL. This study found a negative correlation between YL and LL, a result that corroborated the core trade-offs among leaf functional traits. Long-lived leaves invest substantial amounts of secondary metabolites and structural carbon to enhance stress resistance and prolong their lifespan. However, this reduces N allocation to photosynthetic proteins, resulting in a lower net photosynthetic rate (Shipley et al., 2006). Despite their longer photosynthetic duration, their lower instantaneous carbon assimilation capacity likely results in a lower annual carbon gain per unit leaf area. This is compared to short-lived, high-photosynthetic leaves that employ an acquisitive resource-use strategy. This suggests that achieving high annual carbon fixation relies more on high-intensity instantaneous assimilation than merely on extending leaf lifespan (Qian J. et al., 2025). This further illustrates the intrinsic trade-off plants face between maximizing carbon gain and conserving resources.

By comparing the effects of various leaf functional traits on P_max_ and YL, we found that SLA, SPAD, and LL emerged as key traits affecting the overall photosynthetic carbon fixation in these eight urban shrub species. Rather than acting independently, these traits function through synergies and trade-offs. This shapes adaptive strategies of carbon investment versus carbon return, ultimately driving divergence in photosynthetic carbon fixation capacity (Wright et al., 2001, 2002). Among these, SLA serves as the structural foundation for resource investment, determining leaf construction cost and potential photosynthetic area (Wang et al., 2017). High SLA is typically associated with thinner leaves, higher N_area_, and greater Gs, providing the structural basis for high metabolic activity (Walker et al., 2023). Chl content modulates light capture intensity, especially under non-saturating light. An increase in Chl enhances energy input to the light reactions, efficiently converting CO_2_ diffusion potential into carbon assimilation and supporting higher photosynthetic rates (Ruban and Saccon, 2022). LL represents the temporal dimension of carbon return. Together with SLA and Chl content, it forms a continuum between “fast acquisition-short-term efficiency” and “slow recovery-long-term robustness” strategies. This systematically regulates total leaf-level carbon assimilation. These findings further support that landscape deciduous shrubs can optimize their carbon acquisition strategies through specific combinations of key leaf functional traits. This involves trait synergies and trade-offs that enhance both adaptability and leaf-level photosynthetic carbon fixation.

## 5 Limitations

This study used maximum net photosynthetic rate per unit leaf area (P_max_) and annual carbon fixation per unit leaf area (YL) as indicators of leaf-level photosynthetic carbon fixation. It also preliminarily explored how leaf functional traits affect photosynthetic carbon fixation in landscape deciduous shrubs. Due to limitations of photosynthetic measurement requirements, the tree species and sample size were limited. Future studies could expand the sample size to include more shrub species or incorporate different plant life forms across a broader range of regions. This would help reveal the relationship between leaf functional traits and photosynthetic carbon fixation capacity in urban trees more comprehensively. It would also aid in validating the generalizability of our findings. Furthermore, carbon sinks at the individual plant level involve processes such as photosynthate accumulation and carbon storage. They are also influenced by multiple factors, including plant size, canopy structure, and total leaf area. Future studies could integrate plant carbon storage to assess the effects of functional traits on individual-level carbon sinks more systematically, thereby enhancing their practical value for landscape plant selection.

## 6 Conclusions

This study investigated the leaf functional traits and photosynthetic carbon fixation characteristics of eight common landscape deciduous shrubs in Beijing urban parks. It aimed to elucidate how leaf functional traits affect photosynthetic carbon fixation. We found significant interspecific differentiation in leaf structural, physiological, and chemical traits, as well as in leaf lifespan. This indicates distinct LES strategies among these shrubs. These findings also suggest that the LES applies to carbon fixation strategies of landscape plants. In adapting to urban environments, these deciduous shrubs have developed distinct adaptation and carbon acquisition strategies. Species such as *H. syriacus*, *R. hybrida*, *C. alba*, and *W. florida* exhibited higher SLA, N_area_, P_area_, SPAD, Gs, Tr and PNUE, and tended to adopt an acquisitive resource-use strategy while demonstrating stronger photosynthetic carbon fixation capacity. In contrast, *S. sorbifolia*, *D. parviflora*, *V. opulus*, and *L. maackii* showed greater LT, LMA, LDMC, and longer LL, and tended toward a conservative resource-use strategy with relatively weaker photosynthetic carbon fixation. Our findings reveal that leaf functional traits regulate photosynthetic efficiency and annual carbon assimilation in shrub leaves through synergies and trade-offs. Specifically, SLA, N_area_, P_area_, Gs, Tr, SPAD, and PNUE form a synergistic trait network that enhances photosynthetic carbon fixation. Conversely, LDMC, LMA, and LL reflect plant strategies in structural defense and persistence, exhibiting a clear resource allocation trade-off with the former group. This trade-off strategy illustrates the adaptive compromise plants make between “rapid growth” and “long-term survival”. It leads plants to adopt different carbon acquisition strategies, resulting in differentiated photosynthetic carbon fixation capacities. Among the traits examined, SLA, SPAD, and LL are the key leaf functional traits influencing photosynthetic carbon fixation in these shrubs. Future efforts could prioritize shrub species with large leaf size, high greenness, and long canopy duration, as these traits may indicate stronger photosynthetic carbon fixation capacity. This study provides scientific direction and theoretical support for the selection and breeding of shrub species and new varieties with high carbon fixation capacity.

## Data Availability

The raw data supporting the conclusions of this article will be made available by the authors, without undue reservation.
